# Coupling between bacterial phylogenetic diversity and heterotrophic productivity in a coastal ecosystem affected by estuarine plumes

**DOI:** 10.1093/ismeco/ycaf102

**Published:** 2025-06-20

**Authors:** Yao Liu, Shujie Cai, Wenxin Fan, Wupeng Xiao, Xin Liu, Edward A Laws, Bangqin Huang

**Affiliations:** State Key Laboratory of Marine Environmental Science / National Observation and Research Station for the Taiwan Strait Marine Ecosystem (T-SMART) / Fujian Provincial Key Laboratory for Coastal Ecology and Environmental Studies / College of the Environment and Ecology, Xiamen University, Xiamen, Fujian 361102, China; State Key Laboratory of Marine Environmental Science / National Observation and Research Station for the Taiwan Strait Marine Ecosystem (T-SMART) / Fujian Provincial Key Laboratory for Coastal Ecology and Environmental Studies / College of the Environment and Ecology, Xiamen University, Xiamen, Fujian 361102, China; State Key Laboratory of Marine Environmental Science / National Observation and Research Station for the Taiwan Strait Marine Ecosystem (T-SMART) / Fujian Provincial Key Laboratory for Coastal Ecology and Environmental Studies / College of the Environment and Ecology, Xiamen University, Xiamen, Fujian 361102, China; State Key Laboratory of Marine Environmental Science / National Observation and Research Station for the Taiwan Strait Marine Ecosystem (T-SMART) / Fujian Provincial Key Laboratory for Coastal Ecology and Environmental Studies / College of the Environment and Ecology, Xiamen University, Xiamen, Fujian 361102, China; State Key Laboratory of Marine Environmental Science / National Observation and Research Station for the Taiwan Strait Marine Ecosystem (T-SMART) / Fujian Provincial Key Laboratory for Coastal Ecology and Environmental Studies / College of the Environment and Ecology, Xiamen University, Xiamen, Fujian 361102, China; Department of Environmental Sciences, College of the Coast & Environment, Louisiana State University, Baton Rouge, LA 70803, United States; State Key Laboratory of Marine Environmental Science / National Observation and Research Station for the Taiwan Strait Marine Ecosystem (T-SMART) / Fujian Provincial Key Laboratory for Coastal Ecology and Environmental Studies / College of the Environment and Ecology, Xiamen University, Xiamen, Fujian 361102, China

**Keywords:** bacterioplankton, bacterial productivity, phylogenetic diversity, diversity-productivity relationship, estuarine plumes

## Abstract

Understanding the diversity-productivity relationship (DPR) is crucial for elucidating the ecological functions of marine bacterioplankton. However, studies have often focused on species diversity, neglecting phylogenetic diversity, which may offer deeper insights into the complex ecological processes shaping DPR in natural systems. This study addressed this gap by exploring the role of phylogenetic diversity in bacterioplankton productivity in the northern South China Sea, a coastal ecosystem influenced by estuarine plumes. We aimed to disentangle the mechanisms driving DPR and investigate how estuarine plumes modulate these processes. Our results show that the substantial enhancement of phytoplankton production by the Pearl River plume increased bacterial production, abundance, and cell-specific production. From a metacommunity perspective, phylogenetic diversity, rather than species diversity, significantly enhanced productivity. The plume reduced positive species interactions and complementarity but amplified the selection effect, where increased phylogenetic diversity raised the likelihood of including highly productive species. In plume-impacted communities, distantly related and highly productive clades dominated the DPR. Phylogenetically diverse assemblages exhibited enhanced niche differentiation that facilitated the stable coexistence of productive clades by mitigating exclusion. We also delineated how the negative selection effect and increased species exclusion contributed to the decoupling of species diversity from productivity in communities unaffected and affected by the plume, respectively. These findings highlighted the pivotal role of estuarine plumes in enhancing productivity via increased phylogenetic diversity and in eliciting complex adaptive responses within bacterioplankton communities. Future comprehensive assessments will be needed to elucidate the implications of these dynamics on marine ecosystem services.

## Introduction

Bacterioplankton is a fundamental component of marine ecosystems and represents one of the most diverse and metabolically active microbial groups in the oceans [1, 2]. These microorganisms metabolize a substantial portion of primary production and convert labile dissolved organic matter into biomass through secondary production or bacterial production, thereby forming the cornerstone of the microbial loop [3–5]. As key drivers of nutrient recycling and ecosystem stability, revealing the regulatory mechanisms of their heterotrophic productivity is essential for elucidating their ecological role in marine biogeochemical cycles [6, 7].

The bacterial diversity-productivity relationship (DPR) has been widely explored within the biodiversity-ecosystem functioning framework [8–21]. Biodiversity is multifaceted and influences productivity in two nonexclusive mechanisms: the complementary effect, in which diverse assemblages enhance resource use via positive interactions, and the selection effect, whereby diversity increases the likelihood of including highly productive species [12, 22, 23]. However, most studies have focused on species diversity (taxonomic DPR) while overlooking phylogenetic diversity (phylogenetic DPR). Although artificial bacterial community experiments commonly reported positive correlations between species diversity (e.g. richness) and biomass production [10–12], findings in natural bacterioplankton communities remain inconsistent, showing positive, negative, or no correlation [13–17]—likely due to overlooked functional differences among community members. In contrast, phylogenetic diversity captures evolutionary relationships and provides a more ecologically meaningful perspective, as closely related microorganisms often share similar functional traits and ecological niches, a concept known as phylogenetic niche conservatism [24–27]. Evidence from artificial communities and Mediterranean Sea suggests that bacterial communities composed of more distantly related clades generally exhibit higher productivity [18–21], outperforming species richness alone as a predictor of ecosystem functioning. Despite these insights, research incorporating phylogenetic traits into marine bacterial DPR remains limited, particularly in dynamic coastal ecosystems.

Estuarine plumes are recognized as highly diverse and productive marine environments; they serve as foci of biological activity and play crucial roles in biogeochemical cycling within coastal ecosystems [28, 29]. Nutrient enrichment from estuaries stimulates phytoplankton production and enhances dissolved organic matter availability in coastal waters; the result is a rapid response from bacterioplankton [30]. Studies in typical coastal ecosystems have consistently shown that plumes significantly enhance bacterial productivity and have preliminarily identified copiotrophic bacteria as the dominant contributors based on ecotypic or taxonomic analyses [31–33]. Although detailed reports on the diversity of these copiotrophic bacteria are lacking, a decrease in bacterial species diversity has been observed in low-salinity plumes [34–36]. This pattern indicates a decoupling of productivity from species diversity, implying the need to reassess DPR from a phylogenetic perspective. Furthermore, the mechanisms driving DPR in plume-impacted bacterioplankton communities, particularly the roles of the complementary and selection effects, remain poorly understood.

To address current knowledge gaps, we conducted high-resolution field observations in the coastal waters of the northern South China Sea (SCS), which is significantly impacted by the Pearl River plume [37]. Previous studies have shown that environmental gradients, the composition of the plankton community, and biological activities exhibit remarkable variability from the Pearl River estuary to coastal waters [32, 34, 38, 39]. This region consequently serves as an ideal system to examine DPR. By investigating bacterial production, biomass, and community composition, our objectives were to: (i) clarify the relationship between diversity and productivity in plume-impacted bacterioplankton communities, with a specific focus on phylogenetic DPR; and (ii) explore whether the complementarity or selection effect makes a stronger contribution to the observed DPR, if significant diversity effects are present.

## Materials and methods

### Study area, sampling, and environmental parameters

**Figure 1:**
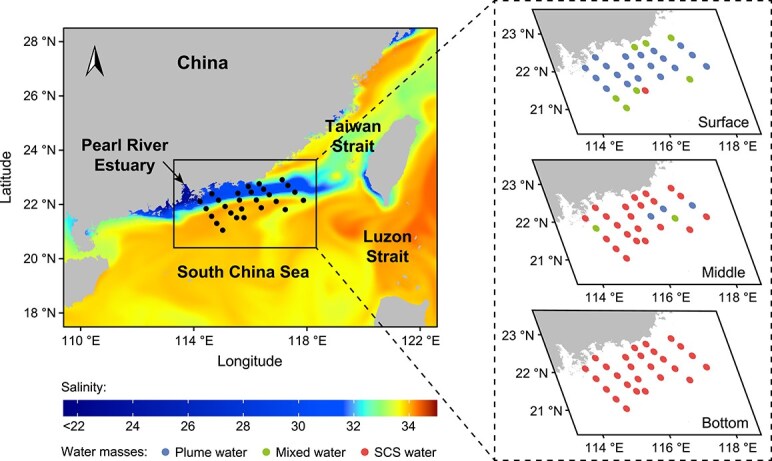
Sampling sites and water mass distribution in the northern SCS. Left panel: sampling sites with an overlaid color gradient showing the average sea surface salinity from 21 July to 26 July 2022, sourced from Copernicus Marine Data (https://data.marine.copernicus.eu/products). Right panels: detailed distribution of different water masses across three depth strata—surface, middle, and bottom—in the study area. The points are colored to represent different water masses: plume water (blue), mixed water (green), and SCS water (red).

A cruise was conducted in the northern SCS from 21 July to 26 July 2022 (Fig. 1 and Supplementary Table S1). Sampling was conducted at 27 stations using 10-l Niskin bottles. A total of 81 water samples were collected from three distinct layers: the surface (3 m below the sea surface), the middle (either at the deep chlorophyll maximum layer, or at the midpoint of the water column if the deep chlorophyll maximum was not clearly defined), and the bottom (3 m above the seafloor). Temperature and salinity were monitored in situ using a SBE9/11 conductivity-temperature-depth recorder (Sea-Bird Electronics, Bellevue, WA, USA). Chlorophyll-*a* concentrations were measured using high-performance liquid chromatography following the method described by Tong *et al.* [38].

Environmental data showed that the water column structure in our study area was strongly influenced by the mixing of seawater and estuarine freshwater inputs (Supplementary Fig. S1A and B). For subsequent analyses comparing bacterial variations across different environments, we divided the region into three water masses following the definitions provided by Wu *et al.* [40]: plume water (salinity <33), SCS water (salinity >33.75), and mixed water with salinity ranging from 33 to 33.75 (Fig. 1 and Supplementary Table S1).

### Bacterial production and abundance

Bacterial production was estimated using the ^3^H-leucine incorporation method [41]. For each sample, four 1.8-ml aliquots of water were incubated for 2 h in the dark at *in situ* temperature with 10 nmol l^−1^ (final concentration) ^3^H-leucine (Perkin Elmer, Boston, MA, USA). One aliquot was immediately fixed with 100 μl of 100% trichloroacetic acid as a control. The remaining three were terminated with trichloroacetic acid at the end of the incubation. The samples were filtered onto 0.2-μm polycarbonate filters (Millipore, Billerica, MA, USA) and washed twice with 4 ml of 5% trichloroacetic acid and 2 ml of 80% ethanol. The filters were then placed in scintillation vials with 4 ml of scintillation solution and left for 24 h. Disintegrations per minute were measured using a Tri-Carb 2800TR liquid scintillation counter (Perkin Elmer, Boston, MA, USA). To convert leucine incorporation into carbon units, we applied an average conversion factor of 0.35 kg C mol leucine^−1^ based on the empirical leucine-to-carbon conversion factors determined by Li *et al.* [42] in the Pearl River Estuary and the northern SCS. Cell-specific bacterial production was calculated as the ratio of production to abundance.

Bacterial abundance was measured to assess bacterial biomass. Samples were collected from 1.8 ml of water, fixed for 15 min in the dark with 0.5% (final concentration) glutaraldehyde. The samples were then stained for 15 min in the dark with 0.5% (final concentration) SYBR Green (Molecular Probes, Eugene, OR, USA) and enumerated using an Accuri C6 flow cytometer (Becton Dickinson, Franklin Lakes, NJ, USA) with 1-μm fluorescent latex beads (Polysciences, Warrington, PA, USA) added for calibration. Final cell numbers were counted based on a bivariate scatter plot of side scatter (related to cell size) and green fluorescence (related to nucleic acid content). Following the definitions provided by Marie *et al.* [43], two ecotypes of bacteria were classified based on the bimodal distribution of nucleic acid content: low nucleic acid (LNA) and high nucleic acid (HNA) (Supplementary Fig. S2). LNA bacteria, typically oligotrophic, are characterized by low productivity, slow growth rates, and high resource affinity. In contrast, HNA bacteria tend to be copiotrophic and are characterized by high productivity, fast growth rates, and lower resource affinity [33].

### Bacterial community composition

To prepare samples for bacterial community analysis, seawater was initially filtered through 200-μm bolting cloth to remove larger organisms. Subsequently, 1–2 l of prefiltered water was filtered onto 0.2-μm polycarbonate filters to capture bacterial cells. Bacterial DNA was extracted from these filters using the FastDNA SPIN Kit for Soil (MP Biomedicals, Santa Ana, CA). The DNA samples were then sent to Genesky Biotechnologies Inc. (Shanghai, China) for polymerase chain reaction amplification of the V3−V4 regions of the bacterial 16S rRNA genes using primers 341F (5′-CCTACGGGNGGCWGCAG-3′) and 805R (5′-GACTACHVGGGTATCTAATCC-3′). The resulting amplicons were sequenced on the Illumina NovaSeq 6000 sequencer.

The raw reads of 16S rRNA gene amplicon sequences were processed in QIIME2 [44]. Adaptor and primer sequences were trimmed using the cutadapt plugin, followed by quality control and identification of amplicon sequence variants (ASVs) using the DADA2 plugin. Taxonomic assignments were made using the Ribosomal Database Project (version 11.5) [45] with a confidence threshold of 0.8 via a pretrained Naive Bayes classifier. ASVs identified as nonheterotrophic bacteria, including mitochondria, chloroplasts, archaea, and cyanobacteria, were excluded from further analysis. Variations in 16S rRNA operon copy numbers were corrected using the *rrn*DB database (version 5.8) [46]. To standardize sampling effort, the ASV table was rarefied to the minimum sequence numbers. Finally, a phylogenetic tree was generated with FastTree plugin.

### Diversity analysis of bacterial communities

All analyses were carried out in R software (version 4.2.0) unless otherwise specified. Species diversity was quantified by ASV-based richness and the Shannon index following standard practices in microbial community studies. To eliminate the covariance between phylogenetic diversity and richness, we computed the abundance-weighted mean pairwise phylogenetic distance (MPD) according to the equation [47]:


(1) \begin{equation*} \mathrm{MPD}=\frac{\sum_{i=1}^n{\sum}_{j\ne i}^n{w}_i{w}_j{d}_{ij}}{\sum_{i=1}^n{\sum}_{j\ne i}^n{w}_i{w}_j} \end{equation*}

here, *n* is the number of ASVs in the phylogenetic tree, *w_i_* and *w_j_* are the abundances of ASV *i* and *j*, respectively, and *d_ij_* is the phylogenetic distance between them. The observed MPD was then compared to the mean MPD from a randomly generated community (null model) and normalized by the standard deviation of the MPD in the null model to calculate its standardized equivalent (SES_MPD_) [47]:


(2) \begin{equation*} {\mathrm{SES}}_{\mathrm{MPD}}=\frac{\mathrm{MPD}-\mathrm{Mean}\ \mathrm{of}\ \mathrm{Null}\ \mathrm{MPD}}{\mathrm{Standard}\ \mathrm{Deviation}\ \mathrm{of}\ \mathrm{Null}\ \mathrm{MPD}} \end{equation*}

The null model randomized the community data matrix using the independent swap algorithm, which maintained the frequency of species occurrence and richness of sample species. The SES_MPD_ was therefore independent of richness and provided a standardized measure of phylogenetic diversity. This measure helps to highlight patterns of phylogenetic diversity that are not confounded by species abundance or community richness, which makes it a valuable metric in studies where richness and diversity are highly variable [19]. Positive SES_MPD_ values suggest a greater phylogenetic distance among co-occurring species and higher phylogenetic diversity than expected by chance, whereas negative values indicate lower phylogenetic diversity than expected by chance.

To explore the relationships between bacterial communities and environmental factors, we conducted a distance-based redundancy analysis (db-RDA) using the “vegan” package [48]. In addition, we used partial least squares path modeling (PLS-PM) from the “plspm” package [49] to assess the interrelationships among bacterial productivity, phylogenetic diversity, and environmental factors. PLS-PM was chosen because it can account for both direct and indirect effects between variables, thereby offering a holistic view of how these factors influence bacterial productivity [50]. PLS-PM was particularly suited for our study as it allows for the quantification of complex interactions in multi-variable systems, such as those present in coastal ecosystems [30].

### Evaluation of complementarity and selection effect

Community-level functional complementarity was assessed by measuring the total branch length of the functional dendrogram of co-occurring species [51]. Since functional dissimilarity in prokaryotes is often linked to genetic distance [24–27], we used the total branch length of the phylogenetic tree as a proxy for complementarity. To infer species co-occurrence, we applied the SparCC algorithm [52] to estimate compositionality-robust correlations across all ASVs with 100 bootstraps. SparCC was selected due to its ability to reduce the bias inherent in compositional data and its robustness in capturing species interactions across complex communities [52]. This method allowed us to generate a meta-community co-occurrence network based on significant correlations (*P* < .01 and absolute correlation coefficient ≥ 0.75). A subnetwork for each sample was generated by preserving ASVs present in each sample using the “subgraph” function in the “igraph” package [53]. The total branch length of the phylogenetic trees of co-occurring ASVs within each subnetwork was then calculated. Meanwhile, we analyzed the connectivity, degree, cohesion, clustering coefficient, and modularity of each subnetwork [53, 54]. These topological features can enhance understanding of complementarity in bacterial communities by providing an interpretation of patterns of species interactions.

To explore the selection effect, which underscores how the influence on ecosystem processes is dominated by highly productive species, we analyzed the emergence of highly productive clades across different environments. Quantitative data on bacterial ecological traits were obtained using abundance-weighted mean strategy values [55]:


(3) \begin{equation*} {\displaystyle \begin{array}{c}\mathrm{Abundance}-\mathrm{weighted}\ \mathrm{mean}\ \mathrm{strategy}=\frac{\sum_{i=1}^n\left({w}_i\times{v}_i\right)}{\sum_{i=1}^n{w}_i}\end{array}} \end{equation*}

where *n* is the total number of samples, *w_i_* is the abundance of species in sample *i*, and *v_i_* is the environmental or functional variable in sample *i*. We calculated two metrics for each ASV: the abundance-weighted mean salinity, which represented the optimal salinity niche along environmental gradients from the plume to the waters of the SCS, and the abundance-weighted mean cell-specific bacterial production as a metric of individual relative productivity. We subsequently evaluated the relationship between abundance-weighted mean salinity and cell-specific bacterial production. Moreover, indicator ASVs for the plume (salinity niche <33), SCS (salinity niche >33.75), and mixed waters (salinity niche ranging from 33 to 33.75) were defined following the division of water masses. The contribution of these indicators to community diversity was then determined using hierarchical partitioning in the “rdacca.hp” package [56].

To further elucidate the relationship between bacterial phylogeny and niche differentiation, we binned the phylogenetic tree with a branch length of 0.1 distance units. Within each phylogenetic bin, the mean phylogenetic distance and the correlation between the mean relative abundance of ASVs and community productivity were computed. Simultaneously, niche overlap indices among ASVs within each phylogenetic bin were determined using the “spaa” package [57], and niche differences were evaluated based on abundance-weighted mean salinity with the “iCAMP” package [58].

### Statistical analysis

We used a Kruskal–Wallis test to compare differences in bacterial productivity, community diversity, and network topology across water masses. Multiple comparisons were corrected using the Benjamini–Hochberg method.

## Results

### Dynamics of bacterial productivity in response to plume

The water column structure in our study area was characterized by a unique estuarine transition zone (Fig. 1 and Supplementary Fig. S1A and B). Low-salinity waters from the Pearl River Estuary flowed northeastward and formed a plume in the surface layer, whereas high-salinity water from the SCS were distributed mainly in the middle and bottom layers. In plume-impacted waters, the productivity of bacterioplankton was significantly enhanced and exhibited a distribution pattern similar to the increase in chlorophyll-*a* concentrations (Supplementary Fig. S1C−F). Specifically, both bacterial production (Kruskal–Wallis test, *P* < .001) and cell-specific bacterial production (Kruskal–Wallis test, *P* < .001) were highest in the plume and mixed waters and lowest in SCS waters (Table 1). We also observed the highest bacterial abundance in plume waters, which was significantly higher than that in both SCS and mixed waters (Kruskal–Wallis test, *P* < .001) (Table 1). However, the difference in bacterial abundance between SCS and mixed waters was not statistically significant.

**Table 1:** Comparison of bacterial productivity and community diversity across water masses (data are shown as the mean ± standard deviation).

**Water masses**	**Productivity**			**Species diversity**		**Phylogenetic diversity**	
	**BP** (mg C m^−3^ d^−1^)	**BA** (×10^5^ cells ml^−1^)	**sBP** (fg C cell^−1^ d^−1^)	**Richness**	**Shannon**	**MPD**	**SES**_**MPD**_
Plume water	2.37 ± 1.52^a^	6.68 ± 4.04^a^	3.81 ± 2.05^a^	320 ± 59^b^	4.45 ± 0.34^b^	0.78 ± 0.04^a^	0.36 ± 1.04^a^
Mixed water	1.74 ± 1.57^a^	7.56 ± 8.47^b^	3.52 ± 2.28^a^	354 ± 52^b^	4.68 ± 0.35^b^	0.76 ± 0.07^a^	−0.05 ± 1.98^a^
SCS water	0.47 ± 0.46^b^	2.53 ± 1.55^b^	1.99 ± 1.78^b^	581 ± 130^a^	5.20 ± 0.32^a^	0.73 ± 0.04^b^	−1.48 ± 1.39^b^

*Note*: Different letters within each column indicate significant differences for each variable at the *P* < .05 significance level. BP, BA, and sBP denote bacterial production, bacterial abundance, and cell-specific bacterial production, respectively.

### Relationships between diversity and productivity in bacterial communities

**Figure 2:**
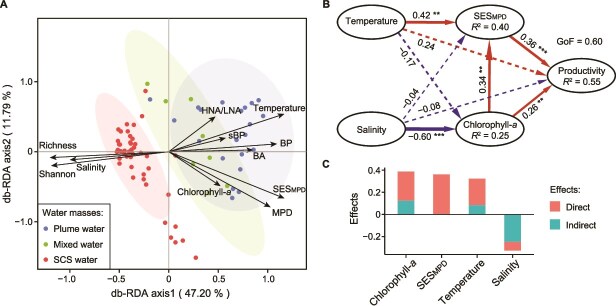
Drivers of bacterial diversity and productivity. (A) db-RDA biplot showing the relationship between bacterial communities and explanatory variables using weighted UniFrac dissimilarity metrics. Points are colored to represent different water masses, and ellipses show the 95% confidence area for the sample coordinates. Arrows in the plot indicate the strength and direction of the effects of each variable. BP, BA, sBP, and HNA/LNA denote bacterial production, bacterial abundance, cell-specific bacterial production, and the ratio of HNA to low nucleic acid bacteria abundance, respectively. (B) Path analysis diagram from PLS-PM illustrating the interrelationships among bacterial productivity, phylogenetic diversity, and key environmental factors. Bacterial productivity is assessed based on bacterial production and bacterial abundance. GoF is the goodness of fit of the model. *R*^2^ indicates the proportion of variance explained by each variable. Arrow width and color are proportional to the standardized path coefficients. Solid and dashed arrows represent significant (*P* < .05) and nonsignificant paths, respectively. Significant levels are: **P < .01 and ***P < .001. (C) Bar chart depicting the direct and indirect effects of each variable on bacterial productivity as determined by PLS-PM.

Bacterial diversity across the three water masses was examined, along with its relationship to bacterial productivity, to better understand the DPR in coastal bacterioplankton communities. Phylogenetic diversity, assessed using MPD and SES_MPD_, was significantly higher in the plume and mixed waters (Kruskal–Wallis test, *P* < .001), whereas species diversity, measured by richness and the Shannon index, peaked in the SCS waters (Kruskal–Wallis test, *P* < .001) (Table 1). The db-RDA revealed distinct shifts in community composition along the salinity gradient (Fig. 2A). With decreasing salinity and concurrent increases in chlorophyll-*a* and temperature, phylogenetic diversity exhibited positive correlations with bacterial production, bacterial abundance, and cell-specific bacterial production, while species diversity became increasingly decoupled from these parameters (Fig. 2A and Supplementary Fig. S3). These findings suggest that plume-driven conditions favor phylogenetically diverse bacterial communities that support enhanced heterotrophic productivity. The PLS-PM further demonstrated that the plume stimulated both bacterial productivity and phylogenetically diverse assemblages through multiple direct and indirect effects (Fig. 2B). Among all tested factors, phylogenetic diversity exerted the strongest direct positive effect on bacterial productivity (Fig. 2C).

### Impact of plume on bacterial co-occurrence network and complementarity

Phylogenetic analysis showed a significant decrease in complementarity from the SCS to plume-impacted bacterial communities (Kruskal–Wallis test, *P* < .001) (Table 2). The co-occurrence network features also indicated that species interactions in the plume and mixed waters generally had a smaller network size (Kruskal–Wallis test, *P* < .001) and connectivity (Kruskal–Wallis test, *P* < .001) compared to those in the SCS waters (Table 2 and Supplementary Fig. S4). Although species interactions in the three water masses were primarily positive, cohesion analysis indicated that the plume significantly increased mutually exclusive interactions (Kruskal–Wallis test, *P* < .001) (Table 2). Lower clustering coefficients (Kruskal–Wallis test, *P* < .001) and higher modularity (Kruskal–Wallis test, *P* < .001) also suggested greater heterogeneity or more independent functional units in plume-impacted waters (Table 2).

**Table 2:** Comparison of topological features and complementarity in bacterial co-occurrence subnetworks across water masses (data are shown as the mean ± standard deviation).

**Water** **masses**	**Network** **Size** **(nodes)**	**Connectivity** **(edges)**	**Degree**	**Positive** **cohesion**	**Negative/** **positive** **cohesion**	**Cluster** **coefficient**	**Modularity**	**Complementarity**
Plume water	45 ± 8^b^	85 ± 27^b^	3.67 ± 0.50^b^	0.35 ± 0.06^b^	0.32 ± 0.14^a^	0.49 ± 0.04^b^	0.59 ± 0.05^a^	8.35 ± 0.60^b^
Mixed water	51 ± 14^b^	108 ± 49^b^	4.07 ± 0.79^b^	0.33 ± 0.04^b^	0.15 ± 0.11^b^	0.54 ± 0.05^a^	0.59 ± 0.04^a^	8.45 ± 0.66^b^
SCS water	83 ± 11^a^	290 ± 50^a^	6.97 ± 0.68^a^	0.40 ± 0.05^a^	0.11 ± 0.03^b^	0.55 ± 0.02^a^	0.43 ± 0.03^b^	9.22 ± 0.49^a^

*Note*: Different letters within each column indicate significant differences for each variable at the *P* < .05 significance level.

### Contributions of key taxa to community diversity and productivity

**Figure 3:**
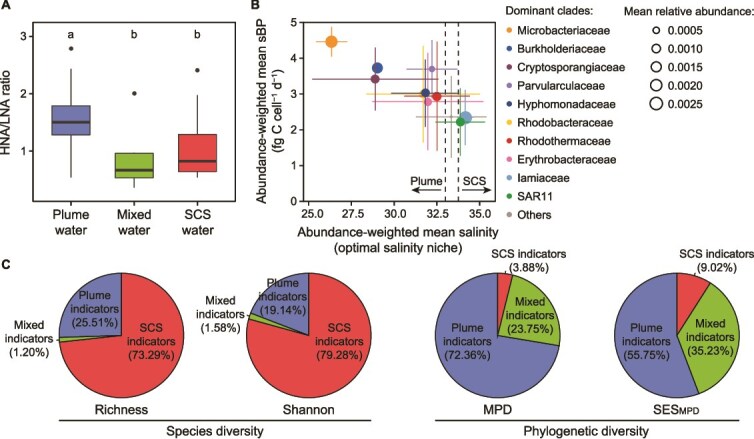
Key bacterial taxa in different water masses. (A) Box plot showing the difference in the ratio of HNA to LNA bacteria abundance among three water masses. Different letters on the boxplot indicate significant differences (*P* < .05). (B) Scatter plot showing the relationship between abundance-weighted mean salinity and abundance-weighted mean cell-specific bacterial production (sBP). The top 10 most abundant families are shown. Values are expressed as mean ± standard deviation based on ASVs included in each family. Points and error bars are colored to represent different families. The size of the points represents mean relative abundance. (C) Pie charts depicting the independent effect of species diversity (richness and the Shannon index) and phylogenetic diversity (MPD and SES_MPD_) within indicators of plume, mixed, and SCS waters on community diversity.

Our analysis revealed distinct patterns in bacterial ecotypes across the three water masses, with significant differences in the composition and productivity of bacterial communities. The SCS waters harbored primarily LNA bacteria, while the plume significantly elevated the proportion of HNA bacteria (Kruskal–Wallis test, *P* < .001) (Fig. 3A). This trend was consistent with the observed increase in both phylogenetic diversity and productivity in bacterial communities (Fig. 2A). Similarly, plume indicators were associated with highly productive and phylogenetically diverse clades (Fig. 3B and C), which determined phylogenetic DPR. The mean relative abundance of highly productive clades, such as Microbacteriaceae, Burkholderiaceae, Cryptosporangiaceae, Parvularculaceae, Hyphomonadaceae, Rhodobacteraceae, and Rhodothermaceae, were positively correlated with bacterial production, bacterial abundance, and cell-specific bacterial production (Supplementary Fig. S5). In contrast, SCS indicators were represented mainly by low-productivity clades such as SAR11 and Iamiaceae (Fig. 3B and Supplementary Fig. S5). Despite the highest species diversity observed among SCS indicators, their contribution to community phylogenetic diversity was minimal (Fig. 3C).

### Balance of phylogenetic relatedness and niche differentiation

**Figure 4:**
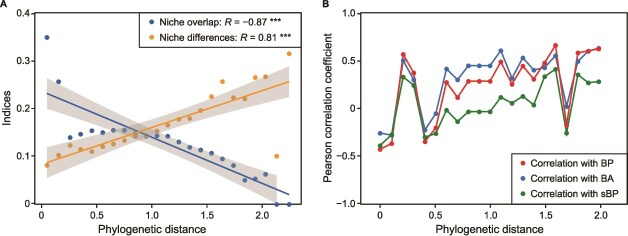
Relationship between bacterial phylogeny and niche dynamics. (A) Scatter plot showing the relationship between mean phylogenetic distance and the indices of mean niche overlap and mean niche differences for ASVs within each phylogenetic bin. *R* indicates the Pearson correlation coefficient. Significant levels are: ***P < .001. (B) Pearson correlation coefficients between mean relative abundance and bacterial production (BP), bacterial abundance (BA), and cell-specific bacterial production (sBP) for ASVs within each phylogenetic bin.

Our analysis revealed a significant trend where increases in phylogenetic distance among ASVs correlated with a decrease in niche overlap and an increase in niche differences (Fig. 4A). The correlation coefficients between the mean relative abundance of ASVs and bacterial production, bacterial abundance, and cell-specific bacterial production shifted from negative or weak to positive as phylogenetic distance increased (Fig. 4B).

## Discussion

### Estuarine plumes enhance phylogenetic diversity and productivity coupling in coastal bacterial communities

Our investigation systematically explored the phylogenetic DPR within coastal bacterioplankton communities influenced by estuarine plumes in the northern SCS. We identified a significant positive correlation between bacterial productivity and phylogenetic diversity, driven by the Pearl River plume, which contrasted with the trends observed in species diversity (Table 1, Fig. 2A, and Supplementary Fig. S3). This positive phylogenetic DPR pattern echoes findings from laboratory and open-ocean studies [18–21], while providing field evidence from a dynamic marginal sea where multiple interacting gradients challenge microbial community stability. Cross-system consistency reinforces a general ecological principle: phylogenetic diversity, by capturing broader evolutionary and functional differentiation, more reliably predicts community productivity than species diversity. This underscores the importance of incorporating phylogenetic perspectives into bacterial DPR assessments, particularly in dynamic coastal ecosystems.

Our results revealed that the growth of copiotrophic bacteria—comprising phylogenetically diverse and highly productive clades—contributed substantially to the enhanced phylogenetic DPR observed in plume-impacted waters (Fig. 3). The suggestion was that these bacteria exploited the enriched substrates provided by the plume, which amplified organic nutrient availability (e.g. phytoplankton production) and created conditions favorable for their rapid proliferation. While copiotrophic bacteria linked to elevated heterotrophic productivity have been reported in other typical estuaries such as those in the Baltic Sea [33] and Mediterranean Sea [31], their phylogenetic composition remains underexplored. Our findings showed that these dominant taxa span a wider range of phylogenetic clades, typically associated with more diverse ecological strategies. For instance, dominant clades such as Microbacteriaceae, Burkholderiaceae, and Rhodothermaceae possess diverse metabolic capabilities that enable them to occupy various niches and thrive in fluctuating nutrient concentrations in estuarine and coastal waters [32, 59–61]. Rhodobacteraceae, another dominant group, possesses both photosynthetic and heterotrophic capabilities [62] gives them an advantage in the sunlit plume waters. Similarly, Hyphomonadaceae, known for their unique particle-attached and epiphytic abilities [63, 64], efficiently utilize organic particle substrates in resource-rich environments. This well-established ecological advantage of copiotrophic bacteria provides a mechanistic explanation for the dominance of highly productive clades and the associated selection effect observed in plume-impacted bacterioplankton communities.

In contrast, lower bacterial productivity and reduced phylogenetic diversity in SCS waters resulted from the dominance of a few oligotrophic clades (Fig. 3). One notable representative was the SAR11 clade, the most abundant oligotrophic bacteria in the oceans, characterized by genome streamlining and specialization in resource utilization—traits that confer advantages in stable and resource-scarce environments [65]. Another dominant clade was Iamiaceae. Information on its biochemical and physiological characteristics is lacking, and its difficulty of isolation implies a dependence on specific environments [66]. The dominance of ecologically similar and slowly growing clades in oligotrophic waters reflects an alternative strategy to that of copiotrophs. These contrasting ecological patterns, shaped by resource availability, demonstrate how estuarine plumes drive trait-environment feedback across diverse phylogenetic lineages.

### The selection effect plays a key role in the phylogenetic DPR of plume-impacted bacterial communities

Further analysis revealed that the selection effect was the predominant driver of phylogenetic DPR in plume-impacted bacterioplankton communities. The growth of copiotrophic bacteria and highly productive clades, which accounted for a significant portion of the phylogenetic diversity and biomass production, vividly illustrated this effect (Fig. 2A and Fig. 3). The dominance of the selection effect has also been confirmed in artificial bacterial communities with lower species richness and phylogenetic relatedness [20]. These findings shed light on how crucial for ecosystem functionality are specific ecological traits of key species within phylogenetically diverse communities. This reinforces the idea that certain trait combinations (e.g. high growth rate, broad metabolic capacity) are repeatedly selected under nutrient-enriched conditions, leading to consistent productivity gains across disparate systems.

The enrichment of copiotrophic bacteria, accompanied by increased species exclusion in plume-impacted waters (Table 2), may have reflected interspecific competition between copiotrophic bacteria and other slowly growing or less common bacteria in resource-rich environments [67]. This dynamic could explain the observed decrease in richness and the decoupling of species diversity from productivity (Table 1, Fig. 2A, and Supplementary Fig. S3). Another consequence of interspecific competition was that the resource niches formed by plumes were occupied by a restricted number of copiotrophic bacteria, accompanied by increased phylogenetic diversity (Fig. 3B and C). The positive selection effect occurs when exclusion processes favor species that strongly influence community productivity, and highly diverse communities are more likely to contain such species [68, 69]. Such selective dominance not only drives biomass accumulation, but also confers functional stability in the face of fluctuating nutrient and physical regimes. The consistency of this evidence again supports the key role of the selection effect in the phylogenetic DPR of plume-impacted bacterial communities.

The complementarity of different species and their positive interactions within bacterial communities were uniformly weakened in the plume-impacted waters (Table 2). This weakening suggested that the dominant copiotrophic bacteria, which thrive in resource-rich environments, can easily access resources and sustain high productivity without extensive cooperation. In contrast, in the resource-scarce SCS waters, greater species synergies were associated with increased complementarity (Table 2). These synergies enabled species to efficiently divide labor and exchange essential metabolites to optimize the use of limited nutrients to sustain basic metabolic functions and stabilize the community [67, 70, 71]. Although increased complementarity theoretically supports higher productivity [20, 23], our findings indicated that such complementarity in oligotrophic conditions prioritizes survival rather than the enhancement of biomass production. The impact of the plume on community productivity thus appeared more direct compared to the more subtle role of complementarity in resource-scarce environments.

In the bacterial communities of the SCS, the highest species diversity was primarily associated with species that were inactive or unproductive (Fig. 3). This pattern suggested a negative-selection effect, wherein highly diverse communities contain more species that do not contribute significantly to productivity [72]. Functions under negative selection often involve narrow phylogenetic constraints, specific metabolic pathways, or energetically expensive reactions [73], which aligns with the characteristics of oligotrophic bacteria. Thus, the negative selection effect reflects the superior survival capacity of more oligotrophic bacteria in resource-scarce environments and plausibly explains the observed decoupling of taxonomic DPR along the environmental gradient from the plume to the offshore waters of the SCS.

Overall, our results revealed that estuarine plumes amplified the selection effect while diminishing the complementarity effect in the phylogenetic DPR of the bacterioplankton communities. In contrast, the lack of an effect between species diversity and productivity in these communities could be attributed to increased species exclusion or the negative-selection effect.

### Phylogenetically diverse assemblages contribute to the coexistence of highly productive clades through niche differentiation

When we extended our analysis of the impact of estuarine plumes on phylogenetic DPR via the selection effect, we also observed intricate adaptive responses within bacterioplankton communities. Communities composed of phylogenetically distant species exhibited greater niche differentiation than those with closely related species (Table 2 and Fig. 4A). This pattern was consistent with the principle of phylogenetic niche conservatism [24–27]. Increased niche differentiation typically sustained a highly productive community structure (Fig. 4B). This observation was consistent with previous research, which indicated that numerous niche differences maximize biomass production by facilitating species coexistence [68].

Biodiversity theories suggest that niche differentiation facilitates species coexistence by enhancing complementarity or weakening exclusion and thereby sustains ecosystem functioning [22, 68, 74, 75]. Our findings revealed that the anticipated complementarity did not significantly enhance productivity. Instead, the heightened species exclusion in plume-impacted waters suggested that niche differentiation served as a mechanism to mitigate this exclusionary pressure (Table 2). Specifically, negative interactions drove the assemblage of phylogenetically distant species, which overcame exclusion by generating greater niche differentiation (e.g. variability in resource utilization). The result was the creation of conditions that favored the coexistence of more highly productive clades. Evidence of this phenomenon has been reported in studies of how estuarine bacteria in distinct groups develop on different dissolved organic carbon substrates with no overlap of dominant groups [76]. These findings illustrate a trait-mediated response to environmental filtering, linking phylogenetic diversity with community reassembly under plume influence.

In addition, DPR may depend on the trophic status of ecosystems. In the oligotrophic northwestern Mediterranean Sea, the complementarity effect is more likely to drive the coupling of phylogenetic diversity and productivity in bacterioplankton communities [19]. Oligotrophic bacteria, such as SAR11, have been shown to dominate bacterial productivity in this region [77]. Given the extensive cooperation among oligotrophic bacteria in the acquisition of resources under oligotrophic conditions [67, 70, 71], we speculated that increased niche differentiation, driven by phylogenetic diversity, formed the foundation of species interdependence and ultimately enhanced secondary production. However, because of the lack of direct evidence, how changes in nutrient availability affect the coupling between diversity and productivity remains to be further elucidated.

## Conclusion

**Figure 5:**
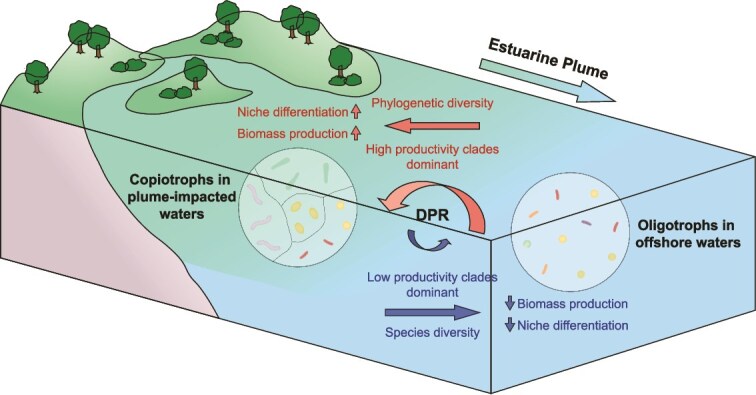
Conceptual diagram illustrating the DPR of bacterioplankton communities in coastal ecosystems under the influence of estuarine plumes. Estuarine plumes expand resource availability in coastal waters, favoring the growth of copiotrophic bacteria with high metabolic activity and productivity. Their dominance enhances overall heterotrophic productivity. Simultaneously, resource-rich conditions intensify competition among copiotrophs, driving niche differentiation within the community. This process selects for the assembly of phylogenetically distant clades with diverse ecological strategies, resulting in a concurrent increase in both productivity and phylogenetic diversity in plume-impacted bacterioplankton communities. In contrast, many oligotrophic taxa—typically less competitive, lower in productivity, and closely related—are excluded from plume waters and tend to persist in resource-scarce environments, where simpler ecological strategies prevail.

Our study presents a novel and systematic perspective on bacterial DPR in coastal ecosystems. We demonstrated that phylogenetic diversity exerts a more substantial role than species diversity in driving heterotrophic productivity in plume-impacted bacterioplankton communities (Fig. 5). This pattern is driven by the dominance of copiotrophic bacteria, which consist of phylogenetically diverse and highly productive clades. This dominance reflects a strong selection effect as the key mechanism underlying the phylogenetic DPR. We also clarify how low phylogenetic relatedness facilitates niche partitioning, enabling the coexistence of these functionally important clades. In addition, the negative selection effect and increased species exclusion accounted for the decoupling of taxonomic DPR in offshore and plume-impacted bacterial communities, respectively. These findings enhance our understanding of how bacterioplankton contribute to coastal ecosystem functioning through evolutionary history and adaptive strategies, and highlight the importance of incorporating phylogenetic traits into biodiversity-ecosystem functioning frameworks. Future large-scale studies are essential to assess how shifts in bacterial diversity impact broader marine ecosystem services under environmental change.

## Supplementary Material

Supplemental_Information_revision_2_ycaf102

## Data Availability

The raw sequencing data are available in the NCBI Sequence Read Archive (SRA) database with the Bioproject accession PRJNA1127221. The scripts used to produce the results in this study are available in the GitHub repository at https://github.com/LiuXYh/northernSCS_2022.

## References

[1] Ibarbalz FM, Henry N, Brandao MC et al. Global trends in marine plankton diversity across kingdoms of life. *Cell* 2019;179:1084–1097.e21. 10.1016/j.cell.2019.10.008 31730851 PMC6912166

[2] Sunagawa S, Coelho LP, Chaffron S et al. Structure and function of the global ocean microbiome. *Science* 2015;348:1261359. 10.1126/science.1261359 25999513

[3] Kujawinski EB . The impact of microbial metabolism on marine dissolved organic matter. *Annu Rev Mar Sci* 2011;3:567–99. 10.1146/annurev-marine-120308-081003 21329217

[4] Azam F . Microbial control of oceanic carbon flux: the plot thickens. *Science* 1998;280:694–6. 10.1126/science.280.5364.694

[5] Fuhrman J . Bacterioplankton roles in cycling of organic matter: The microbial food web. In: Falkowski PG, Woodhead AD, Vivirito K (eds.). Primary Productivity and Biogeochemical Cycles in the Sea. Boston, MA: Springer, 1992, 361–83. 10.1007/978-1-4899-0762-2_20.

[6] Heneghan RF, Holloway-Brown J, Gasol JM et al. The global distribution and climate resilience of marine heterotrophic prokaryotes. *Nat Commun* 2024;15:6943. 10.1038/s41467-024-50635-z 39138161 PMC11322184

[7] Hutchins DA, Fu F. Microorganisms and ocean global change. *Nat Microbiol* 2017;2:17058. 10.1038/nmicrobiol.2017.58 28540925

[8] Mao Z, Zhao Z, Da J et al. The selection of copiotrophs may complicate biodiversity-ecosystem functioning relationships in microbial dilution-to-extinction experiments. *Environmental Microbiome* 2023;18:19. 10.1186/s40793-023-00478-w 36932455 PMC10024408

[9] Lei J, Feng J, Ding J et al. Revisiting the classical biodiversity-ecosystem functioning and stability relationships in microbial microcosms. *PNAS Nexus* 2025;4:pgaf114. 10.1093/pnasnexus/pgaf114 40303002 PMC12038814

[10] Gravel D, Bell T, Barbera C et al. Experimental niche evolution alters the strength of the diversity-productivity relationship. *Nature* 2011;469:89–92. 10.1038/nature09592 21131946

[11] Bell T, Newman JA, Silverman BW et al. The contribution of species richness and composition to bacterial services. *Nature* 2005;436:1157–60. 10.1038/nature03891 16121181

[12] Hodgson DJ, Rainey PB, Buckling A. Mechanisms linking diversity, productivity and invasibility in experimental bacterial communities. *Proc R Soc B* 2002;269:2277–83. 10.1098/rspb.2002.2146 PMC1691149 12427320

[13] Baumas CMJ, Le Moigne FAC, Garel M et al. Mesopelagic microbial carbon production correlates with diversity across different marine particle fractions. *ISME J* 2021;15:1695–708. 10.1038/s41396-020-00880-z 33452475 PMC8163737

[14] Schmidt ML, Biddanda BA, Weinke AD et al. Microhabitats are associated with diversity-productivity relationships in freshwater bacterial communities. *FEMS Microbiol Ecol* 2020;96:fiaa029. 10.1093/femsec/fiaa029 32105331 PMC8453396

[15] Obernosterer I, Lami R, Larcher M et al. Linkage between bacterial carbon processing and the structure of the active bacterial community at a coastal site in the NW Mediterranean Sea. *Microb Ecol* 2010;59:428–35. 10.1007/s00248-009-9588-7 19789909

[16] Danovaro R, Pusceddu A. Biodiversity and ecosystem functioning in coastal lagoons: does microbial diversity play any role? *Estuar Coast Shelf Sci* 2007;75:4–12. 10.1016/j.ecss.2007.02.030

[17] Reinthaler T, Winter C, Herndl GJ. Relationship between bacterioplankton richness, respiration, and production in the southern North Sea. *Appl Environ Microbiol* 2005;71:2260–6. 10.1128/AEM.71.5.2260-2266.2005 15870310 PMC1087554

[18] Roger F, Bertilsson S, Langenheder S et al. Effects of multiple dimensions of bacterial diversity on functioning, stability and multifunctionality. *Ecology* 2016;97:2716–28. 10.1002/ecy.1518 27859115

[19] Galand PE, Salter I, Kalenitchenko D. Ecosystem productivity is associated with bacterial phylogenetic distance in surface marine waters. *Mol Ecol* 2015;24:5785–95. 10.1111/mec.13347 26289961

[20] Venail PA, Vives MJ. Phylogenetic distance and species richness interactively affect the productivity of bacterial communities. *Ecology* 2013;94:2529–36. 10.1890/12-2002.1 24400504

[21] Tan J, Pu Z, Ryberg WA et al. Species phylogenetic relatedness, priority effects, and ecosystem functioning. *Ecology* 2012;93:1164–72. 10.1890/11-1557.1 22764502

[22] Wang S, Hong P, Adler PB et al. Towards mechanistic integration of the causes and consequences of biodiversity. *Trends Ecol Evol* 2024;39:689–700. 10.1016/j.tree.2024.02.008 38503639

[23] Loreau M, Hector A. Partitioning selection and complementarity in biodiversity experiments. *Nature* 2001;412:72–6. 10.1038/35083573 11452308

[24] Davies TJ . Ecophylogenetics redux. *Ecol Lett* 2021;24:1073–88. 10.1111/ele.13682 33565697

[25] Goberna M, Verdu M. Predicting microbial traits with phylogenies. *ISME J* 2016;10:959–67. 10.1038/ismej.2015.171 26371406 PMC4796935

[26] Martiny AC, Treseder K, Pusch G. Phylogenetic conservatism of functional traits in microorganisms. *ISME J* 2013;7:830–8. 10.1038/ismej.2012.160 23235290 PMC3603392

[27] Srivastava DS, Cadotte MW, MacDonald AA et al. Phylogenetic diversity and the functioning of ecosystems. *Ecol Lett* 2012;15:637–48. 10.1111/j.1461-0248.2012.01795.x 22583836

[28] Xenopoulos MA, Downing JA, Kumar MD et al. Headwaters to oceans: ecological and biogeochemical contrasts across the aquatic continuum. *Limnol Oceanogr* 2017;62:S3–14. 10.1002/lno.10721

[29] Bianchi TS . Biogeochemistry of Estuaries. New York, NY: Oxford University Press, 2007, 10.1093/oso/9780195160826.001.0001.

[30] Crump BC, Bowen JL. The microbial ecology of estuarine ecosystems. *Annu Rev Mar Sci* 2024;16:335–60. 10.1146/annurev-marine-022123-101845 37418833

[31] Tomaš AV, Šantić D, Šolić M et al. Dynamics of aerobic anoxygenic phototrophs along the trophic gradient in the central Adriatic Sea. *Deep-Sea Res II Top Stud Oceanogr* 2019;164:112–21. 10.1016/j.dsr2.2019.06.001

[32] Xu J, Li X, Shi Z et al. Bacterial carbon cycling in the river plume in the northern South China Sea during summer. *J Geophys Res Oceans* 2018;123:8106–21. 10.1029/2018jc014277

[33] Kaartokallio H, Asmala E, Autio R et al. Bacterial production, abundance and cell properties in boreal estuaries: relation to dissolved organic matter quantity and quality. *Aquat Sci* 2015;78:525–40. 10.1007/s00027-015-0449-9

[34] Sun P, Wang Y, Huang X et al. Water masses and their associated temperature and cross-domain biotic factors co-shape upwelling microbial communities. *Water Res* 2022;215:118274. 10.1016/j.watres.2022.118274 35298994

[35] Doherty M, Yager PL, Moran MA et al. Bacterial biogeography across the amazon river-ocean continuum. *Front Microbiol* 2017;8:882. 10.3389/fmicb.2017.00882 28588561 PMC5440517

[36] Fortunato CS, Herfort L, Zuber P et al. Spatial variability overwhelms seasonal patterns in bacterioplankton communities across a river to ocean gradient. *ISME J* 2012;6:554–63. 10.1038/ismej.2011.135 22011718 PMC3280145

[37] Ou S, Zhang H, Wang D et al. Horizontal characteristics of buoyant plume off the pearl river estuary during summer. *J Coast Res* 2024;50:652–7. 10.2112/JCR-SI50-123.1

[38] Tong Z, Ma L, Cai S et al. Responses of phytoplankton communities to the effect of both river plume and coastal upwelling. *Journal of geophysical research*. *Biogeosciences* 2023;128:e2023JG007486. 10.1029/2023jg007486

[39] Wu W, Liu H. Disentangling protist communities identified from DNA and RNA surveys in the Pearl River-South China Sea continuum during the wet and dry seasons. *Mol Ecol* 2018;27:4627–40. 10.1111/mec.14867 30222225

[40] Wu K, Dai M, Li X et al. Dynamics and production of dissolved organic carbon in a large continental shelf system under the influence of both river plume and coastal upwelling. *Limnol Oceanogr* 2017;62:973–88. 10.1002/lno.10479

[41] Kirchman D . Measuring bacterial biomass production and growth rates from leucine incorporation in natural aquatic environments. *Methods Microbiol* 2001;**30**:227–37. 10.1016/S0580-9517(01)30047-8.

[42] Li X, Xu J, Shi Z et al. Variability in the empirical leucine-to-carbon conversion factors along an environmental gradient. *Acta Oceanol Sin* 2018;37:77–82. 10.1007/s13131-018-1144-1

[43] Marie D, Simon N, Vaulot D. Phytoplankton cell counting by flow cytometry. *Algal Culturing Techniques* 2005;**1**:253–67. 10.1016/B978-012088426-1/50018-4

[44] Bolyen E, Rideout JR, Dillon MR et al. Author correction: reproducible, interactive, scalable and extensible microbiome data science using QIIME 2. *Nat Biotechnol* 2019;37:1091. 10.1038/s41587-019-0252-6 31399723

[45] Cole JR, Wang Q, Fish JA et al. Ribosomal database project: data and tools for high throughput rRNA analysis. *Nucleic Acids Res* 2014;42:D633–42. 10.1093/nar/gkt1244 24288368 PMC3965039

[46] Stoddard SF, Smith BJ, Hein R et al. rrnDB: improved tools for interpreting rRNA gene abundance in bacteria and archaea and a new foundation for future development. *Nucleic Acids Res* 2015;43:D593–8. 10.1093/nar/gku1201 25414355 PMC4383981

[47] Webb CO, Ackerly DD, Kembel SW. Phylocom: software for the analysis of phylogenetic community structure and trait evolution. *Bioinformatics* 2008;24:2098–100. 10.1093/bioinformatics/btn358 18678590

[48] Oksanen J, Blanchet FG, Kindt R et al. Package ‘vegan’. In: Community Ecology Package, Version 2017, 2.

[49] Sanchez G . PLS Path Modeling with R, Vol. 383. Berkeley: Trowchez Editions, 2013, 551.

[50] Zeng N, Liu Y, Gong P et al. Do right PLS and do PLS right: a critical review of the application of PLS-SEM in construction management research. *Front Eng Manag* 2021;8:356–69. 10.1007/s42524-021-0153-5

[51] Devoto M, Bailey S, Craze P et al. Understanding and planning ecological restoration of plant-pollinator networks. *Ecol Lett* 2012;15:319–28. 10.1111/j.1461-0248.2012.01740.x 22251948

[52] Friedman J, Alm EJ. Inferring correlation networks from genomic survey data. *PLoS Comput Biol* 2012;8:e1002687. 10.1371/journal.pcbi.1002687 23028285 PMC3447976

[53] Csardi G, Nepusz T. The igraph software. *Complex Systems* 2006;1695:1–9.

[54] Herren CM, McMahon KD. Cohesion: a method for quantifying the connectivity of microbial communities. *ISME J* 2017;11:2426–38. 10.1038/ismej.2017.91 28731477 PMC5649174

[55] Massing JC, Fahimipour AK, Bunse C et al. Quantification of metabolic niche occupancy dynamics in a Baltic Sea bacterial community. *mSystems* 2023;8:e00028–3. 10.1128/msystems.00028-23 37255288 PMC10312292

[56] Lai J, Zou Y, Zhang J et al. Generalizing hierarchical and variation partitioning in multiple regression and canonical analyses using the rdacca.Hp R package. *Methods Ecol Evol* 2022;13:782–8. 10.1111/2041-210x.13800

[57] Zhang J, Zhang MJ. Package ‘spaa’. In: R Package Version 2013, 1.

[58] Ning D, Yuan M, Wu L et al. A quantitative framework reveals ecological drivers of grassland microbial community assembly in response to warming. *Nat Commun* 2020;11:4717. 10.1038/s41467-020-18560-z 32948774 PMC7501310

[59] Liew KJ, Teo SC, Shamsir MS et al. Complete genome sequence of Rhodothermaceae bacterium RA with cellulolytic and xylanolytic activities. *3 Biotech* 2018;8:376. 10.1007/s13205-018-1391-z PMC6087703 30105201

[60] Haggerty JM, Dinsdale EA. Distinct biogeographical patterns of marine bacterial taxonomy and functional genes. *Glob Ecol Biogeogr* 2016;26:177–90. 10.1111/geb.12528

[61] Jeffries TC, Schmitz Fontes ML, Harrison DP et al. Bacterioplankton dynamics within a large anthropogenically impacted urban estuary. *Front Microbiol* 2015;6:1438. 10.3389/fmicb.2015.01438 26858690 PMC4726783

[62] Simon M, Scheuner C, Meier-Kolthoff JP et al. Phylogenomics of rhodobacteraceae reveals evolutionary adaptation to marine and non-marine habitats. *ISME J* 2017;11:1483–99. 10.1038/ismej.2016.198 28106881 PMC5437341

[63] Catao CPE, Pollet T, Garnier C et al. Temperate and tropical coastal waters share relatively similar microbial biofilm communities while free-living or particle-attached communities are distinct. *Mol Ecol* 2021;30:2891–904. 10.1111/mec.15929 33887078

[64] Comba Gonzalez NB, Nino Corredor AN, Lopez Kleine L et al. Temporal changes of the epiphytic bacteria community from the marine macroalga ulva lactuca (Santa Marta, colombian-caribbean). *Curr Microbiol* 78:534–43. 10.1007/s00284-020-02302-x 33388936

[65] Giovannoni SJ . SAR11 bacteria: the most abundant plankton in the oceans. *Annu Rev Mar Sci* 2017;9:231–55. 10.1146/annurev-marine-010814-015934 27687974

[66] Hu D, Cha G, Gao B. A phylogenomic and molecular markers based analysis of the class *Acidimicrobiia*. *Front Microbiol* 2018;9:987. 10.3389/fmicb.2018.00987 29867887 PMC5962788

[67] Dai T, Wen D, Bates CT et al. Nutrient supply controls the linkage between species abundance and ecological interactions in marine bacterial communities. *Nat Commun* 2022;13:175. 10.1038/s41467-021-27857-6 35013303 PMC8748817

[68] Godoy O, Gomez-Aparicio L, Matias L et al. An excess of niche differences maximizes ecosystem functioning. *Nat Commun* 2020;11:4180. 10.1038/s41467-020-17960-5 32826915 PMC7442808

[69] Hooper DU, Chapin FS, Ewel JJ et al. Effects of biodiversity on ecosystem functioning: a consensus of current knowledge. *Ecol Monogr* 2005;75:3–35. 10.1890/04-0922

[70] Calatayud J, Andivia E, Escudero A et al. Positive associations among rare species and their persistence in ecological assemblages. *Nature Ecology & Evolution* 2020;4:40–5. 10.1038/s41559-019-1053-5 31844189

[71] Gandhi SR, Korolev KS, Gore J. Cooperation mitigates diversity loss in a spatially expanding microbial population. *Proc Natl Acad Sci* 2019;116:23582–7. 10.1073/pnas.1910075116 31591225 PMC6876198

[72] Jiang L . Negative selection effects suppress relationships between bacterial diversity and ecosystem functioning. *Ecology* 2007;88:1075–85. 10.1890/06-1556 17536392

[73] Cuellar-Gempeler C . Diversity-function relationships and the underlying ecological mechanisms in host-associated microbial communities. In: Hurst CJ (ed.). Microbes: The Foundation Stone of the Biosphere. Cham: Springer, 2021, 297–326. 10.1007/978-3-030-63512-1_17.

[74] Bulleri F, Bruno JF, Silliman BR et al. Facilitation and the niche: implications for coexistence, range shifts and ecosystem functioning. *Funct Ecol* 2015;30:70–8. 10.1111/1365-2435.12528

[75] Chesson P . Mechanisms of maintenance of species diversity. *Annu Rev Ecol Evol Syst* 2000;31:343–66. 10.1146/annurev.ecolsys.31.1.343

[76] Covert JS, Moran MA. Molecular characterization of estuarine bacterial communities that use high- and low-molecular weight fractions of dissolved organic carbon. *Aquat Microb Ecol* 2001;25:127–39. 10.3354/ame025127

[77] Laghdass M, Catala P, Caparros J et al. High contribution of SAR11 to microbial activity in the north West Mediterranean Sea. *Microb Ecol* 2012;63:324–33. 10.1007/s00248-011-9915-7 21887519

