# Supplementary material for: Coupling between bacterial phylogenetic diversity and heterotrophic productivity in a coastal ecosystem affected by estuarine plumes
Source: ISME Commun. 2025 Jun 20;5(1):ycaf102. doi: 10.1093/ismeco/ycaf102 (PMC12271574; doi:10.1093/ismeco/ycaf102)
Supplement: Supplemental_Information_revision_2_ycaf102 [file supplemental_information_revision_2_ycaf102.pdf]

Supplemental Information for:

Coupling between bacterial phylogenetic diversity and heterotrophic productivity in a coastal ecosystem affected by estuarine plumes

Yao Liu[1], Shujie Cai[1], Wenxin Fan[1], Wupeng Xiao[1,*], Xin Liu[1], Edward A. Laws[2], Bangqin Huang[1,*]

[1] *State Key Laboratory of Marine Environmental Science / National Observation and Research Station for the Taiwan Strait Marine Ecosystem (T-SMART) / Fujian Provincial Key Laboratory for Coastal Ecology and Environmental Studies / College of the Environment and Ecology, Xiamen University, Xiamen 361102, China.*

[2] *Department of Environmental Sciences, College of the Coast & Environment, Louisiana State University, Baton Rouge, Louisiana 70803, USA.*

[*] Corresponding author:

Wupeng Xiao: College of the Environment and Ecology, Xiamen University, Xiamen 361102, China / E-mail: wpxiao@xmu.edu.cn

Table of Contents:

Files	Page
Supplementary Table S1	2
Supplementary Figure S1	5
Supplementary Figure S2	6
Supplementary Figure S3	7
Supplementary Figure S4	8
Supplementary Figure S5	9

Supplementary Table S1 Sampling information of waters collected in the present study.

Sampling station	Longitude (°E)	Latitude (°N)	Sampling depth (m)	Sampling date	Temperature (°C)	Salinity	Water masses
E609	117.85	22.14	3	2022-07-21	29.71	32.47	Plume
E609	117.85	22.14	40	2022-07-21	25.67	34.02	SCS
E609	117.85	22.14	60	2022-07-21	22.37	34.31	SCS
E607	117.55	22.44	3	2022-07-22	30.47	31.26	Plume
E607	117.55	22.44	15	2022-07-22	29.86	31.88	Plume
E607	117.55	22.44	30	2022-07-22	24.93	34.05	SCS
E605	117.30	22.69	3	2022-07-22	31.12	30.35	Plume
E605	117.30	22.69	15	2022-07-22	23.35	34.25	SCS
E605	117.30	22.69	35	2022-07-22	23.25	34.23	SCS
E603	117.10	22.90	3	2022-07-22	28.97	33.32	Mixed
E603	117.10	22.90	15	2022-07-22	22.52	34.32	SCS
E603	117.10	22.90	35	2022-07-22	22.18	34.31	SCS
E501	116.29	22.75	3	2022-07-22	26.64	33.44	Mixed
E501	116.29	22.75	10	2022-07-22	23.07	34.24	SCS
E501	116.29	22.75	29	2022-07-22	21.73	34.34	SCS
E503	116.44	22.55	3	2022-07-22	29.78	27.62	Plume
E503	116.44	22.55	20	2022-07-22	25.40	33.99	SCS
E503	116.44	22.55	37	2022-07-22	21.89	34.35	SCS
E505	116.64	22.35	3	2022-07-23	31.01	25.70	Plume
E505	116.64	22.35	10	2022-07-23	29.60	31.27	Plume
E505	116.64	22.35	40	2022-07-23	22.55	34.32	SCS
E507	116.89	22.10	3	2022-07-23	30.69	32.49	Plume
E507	116.89	22.10	25	2022-07-23	29.78	33.20	Mixed
E507	116.89	22.10	70	2022-07-23	20.74	34.44	SCS
E509	117.21	21.80	3	2022-07-23	30.39	33.34	Mixed
E509	117.21	21.80	75	2022-07-23	24.69	34.20	SCS
E509	117.21	21.80	150	2022-07-23	18.15	34.60	SCS
E407	116.36	21.87	3	2022-07-23	30.97	30.10	Plume
E407	116.36	21.87	60	2022-07-23	25.42	34.07	SCS
E407	116.36	21.87	88	2022-07-23	20.13	34.47	SCS
E405	116.19	22.16	3	2022-07-23	30.99	25.58	Plume
E405	116.19	22.16	10	2022-07-23	30.18	32.15	Plume

E405	116.19	22.16	63	2022-07-23	20.35	34.48	SCS
E403	116.01	22.44	3	2022-07-23	30.98	25.47	Plume
E403	116.01	22.44	25	2022-07-23	23.76	34.15	SCS
E403	116.01	22.44	37	2022-07-23	20.52	34.45	SCS
E401	115.91	22.65	3	2022-07-24	27.05	33.42	Mixed
E401	115.91	22.65	12	2022-07-24	23.91	34.09	SCS
E401	115.91	22.65	23	2022-07-24	21.46	34.39	SCS
E303	115.53	22.40	3	2022-07-24	29.72	29.44	Plume
E303	115.53	22.40	22	2022-07-24	23.11	34.26	SCS
E303	115.53	22.40	33	2022-07-24	22.12	34.33	SCS
E305	115.59	22.15	3	2022-07-24	31.41	26.60	Plume
E305	115.59	22.15	50	2022-07-24	24.05	34.17	SCS
E305	115.59	22.15	60	2022-07-24	20.26	34.47	SCS
E307	115.67	21.83	3	2022-07-24	31.23	32.23	Plume
E307	115.67	21.83	50	2022-07-24	24.18	34.15	SCS
E307	115.67	21.83	93	2022-07-24	19.32	34.50	SCS
E309	115.75	21.50	3	2022-07-24	30.16	33.79	SCS
E309	115.75	21.50	75	2022-07-24	22.69	34.32	SCS
E309	115.75	21.50	110	2022-07-24	19.30	34.50	SCS
E209	115.49	21.50	3	2022-07-24	30.70	33.55	Mixed
E209	115.49	21.50	65	2022-07-24	22.85	34.33	SCS
E209	115.49	21.50	107	2022-07-24	19.48	34.48	SCS
E207	115.29	21.68	3	2022-07-25	30.64	32.76	Plume
E207	115.29	21.68	75	2022-07-25	22.84	34.28	SCS
E207	115.29	21.68	95	2022-07-25	20.12	34.46	SCS
E205	115.08	21.92	3	2022-07-25	30.92	31.37	Plume
E205	115.08	21.92	50	2022-07-25	23.96	34.20	SCS
E205	115.08	21.92	67	2022-07-25	21.50	34.37	SCS
E203	114.85	22.15	3	2022-07-25	31.52	27.62	Plume
E203	114.85	22.15	22	2022-07-25	25.86	33.92	SCS
E203	114.85	22.15	40	2022-07-25	21.43	34.37	SCS
E201	114.62	22.38	3	2022-07-25	26.03	32.99	Plume
E201	114.62	22.38	10	2022-07-25	24.06	34.10	SCS
E201	114.62	22.38	20	2022-07-25	21.82	34.35	SCS
E101	114.2	22.10	3	2022-07-25	31.46	22.08	Plume

E101	114.2	22.10	15	2022-07-25	24.57	34.08	SCS
E101	114.2	22.10	26	2022-07-25	23.35	34.22	SCS
E103	114.41	21.84	3	2022-07-25	31.79	22.20	Plume
E103	114.41	21.84	20	2022-07-25	28.40	33.64	Mixed
E103	114.41	21.84	45	2022-07-25	20.87	34.42	SCS
E105	114.61	21.55	3	2022-07-25	30.81	31.91	Plume
E105	114.61	21.55	50	2022-07-25	23.61	34.22	SCS
E105	114.61	21.55	68	2022-07-25	20.86	34.40	SCS
E107	114.79	21.29	3	2022-07-25	30.60	33.62	Mixed
E107	114.79	21.29	64	2022-07-25	21.86	34.34	SCS
E107	114.79	21.29	87	2022-07-25	21.24	34.36	SCS
E109	115.00	21.04	3	2022-07-26	30.30	33.67	Mixed
E109	115.00	21.04	75	2022-07-26	22.88	34.25	SCS
E109	115.00	21.04	93	2022-07-26	19.09	34.50	SCS

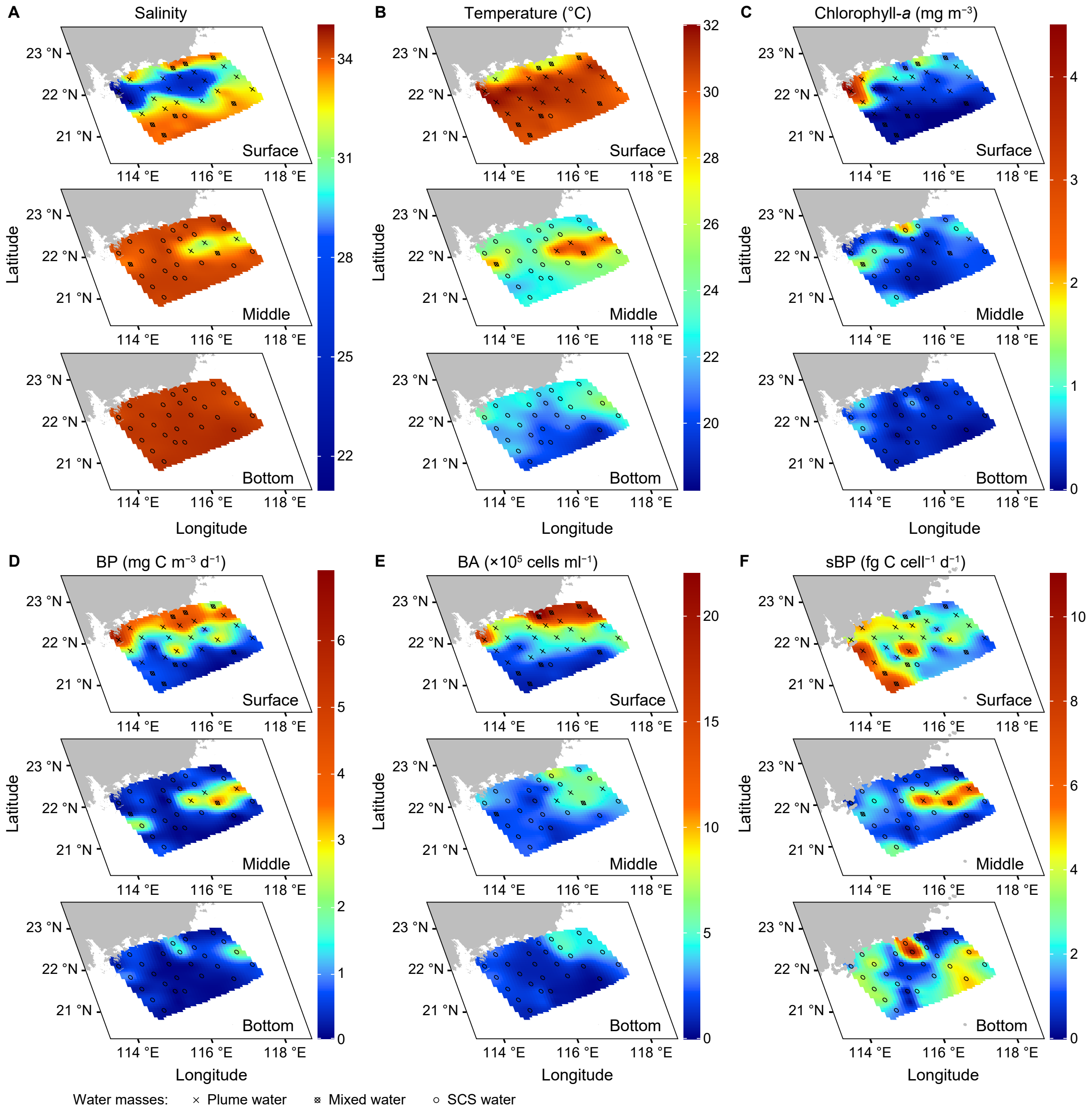

Supplementary Figure S1 Distribution of **A** salinity, **B** temperature, **C** chlorophyll-*a* concentrations, **D** bacterial production (BP), **E** bacterial abundance (BA), and **F** cell-specific bacterial production (sBP) across depth strata in the present study.

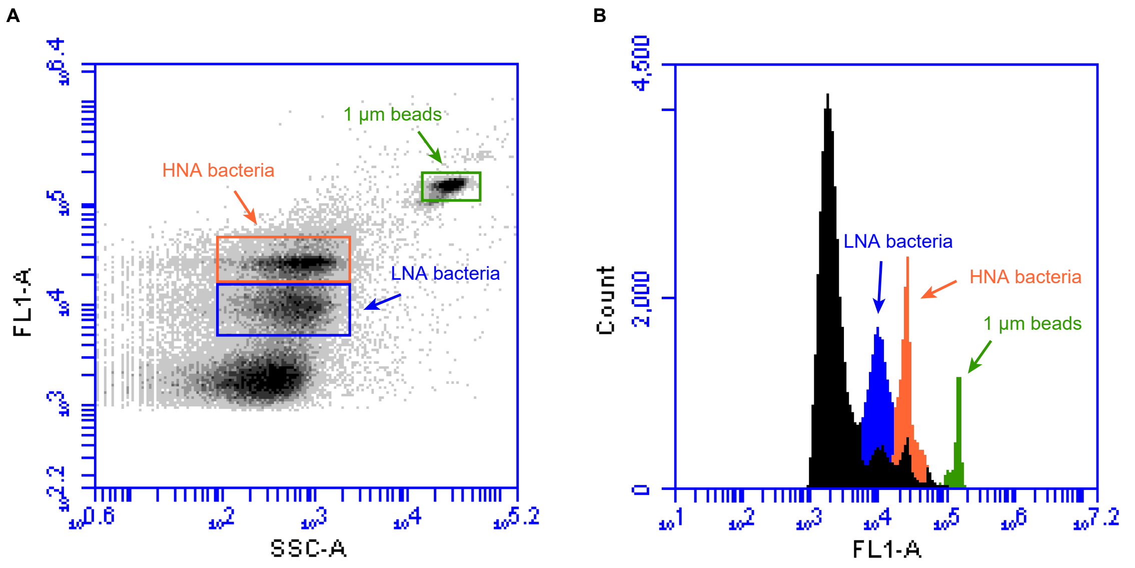

Supplementary Figure S2 Bacterial abundance counting based on flow cytometry. **A** Scatter plot of side scatter (SSC) and green fluorescence (FL1). **B** Frequency distribution of green fluorescence. Images are generated using the Accuri C6 software. Two ecotypes of bacteria, low nucleic acid (LNA) and high nucleic acid (HNA), are classified based on the bimodal distribution of green fluorescence. Total bacterial abundance is the sum of HNA and LNA cells.

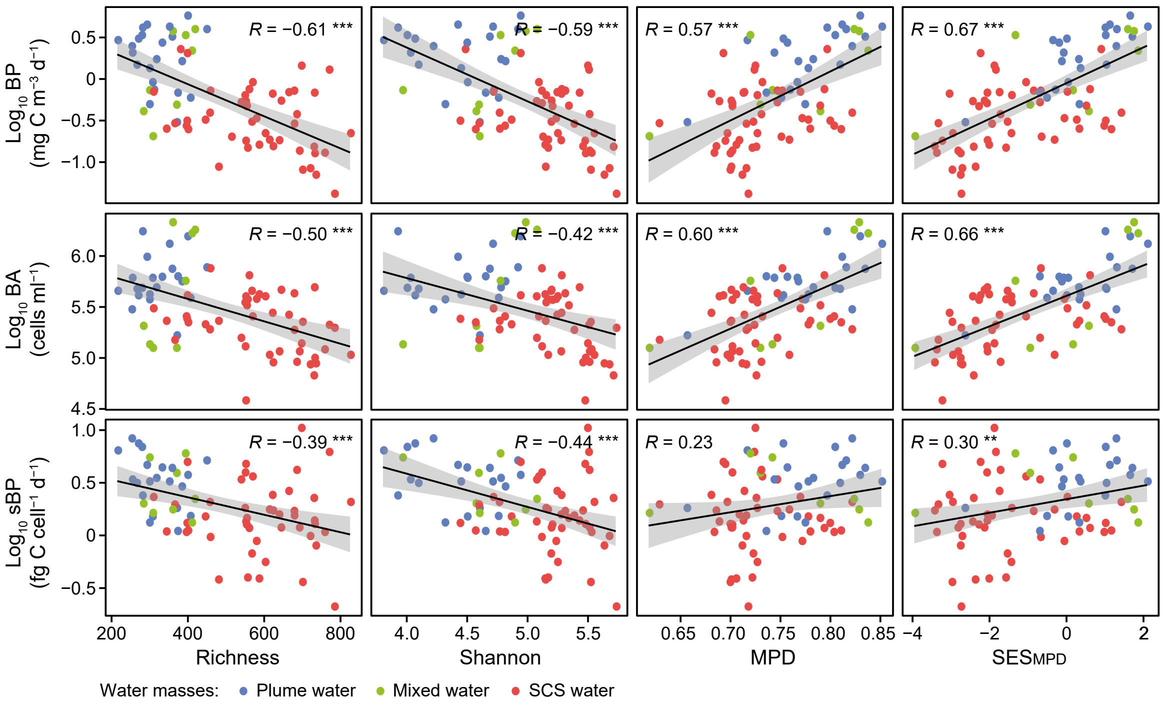

Supplementary Figure S3 Scatter plot showing the relationships between species diversity (richness and the Shannon index) and phylogenetic diversity (MPD and SES_{MPD}) with bacterial production (BP), bacterial abundance (BA), and cell-specific bacterial production (sBP). Points are colored to represent different water masses. *R* indicates the Pearson correlation coefficient. Significant levels are: $^{**}P < 0.01$, and $^{***}P < 0.001$.

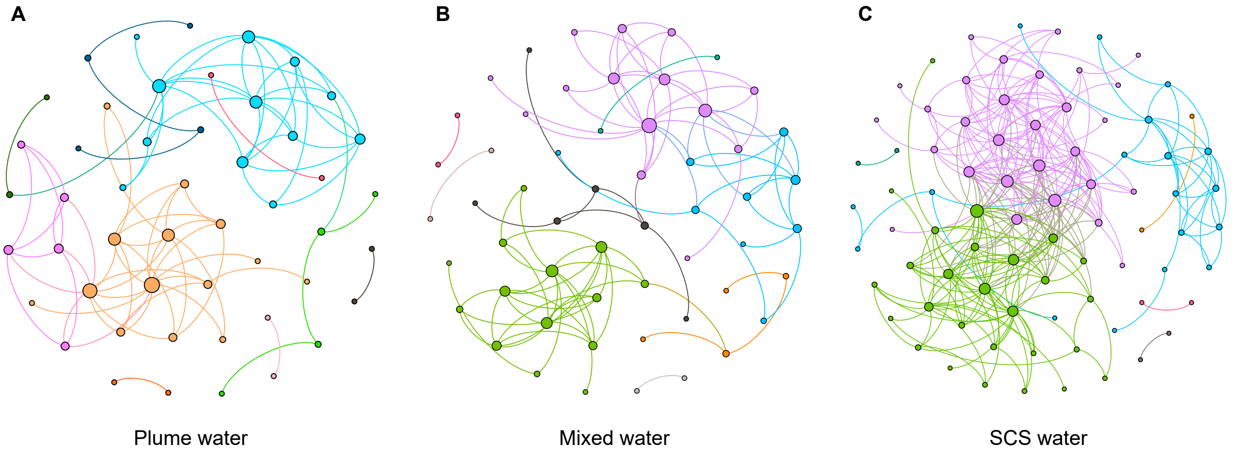

Supplementary Figure S4 Three representative bacterial co-occurrence subnetworks for **A** plume, **B** mixed, and **C** SCS waters are illustrated. Images are generated using the Gephi software. Nodes within the subnetworks represent amplicon sequence variants (ASVs) with significant correlations in the bacterial communities, while edges represent interactions between ASVs. The size of the nodes is proportional to their degree, and different colors indicate distinct modules.

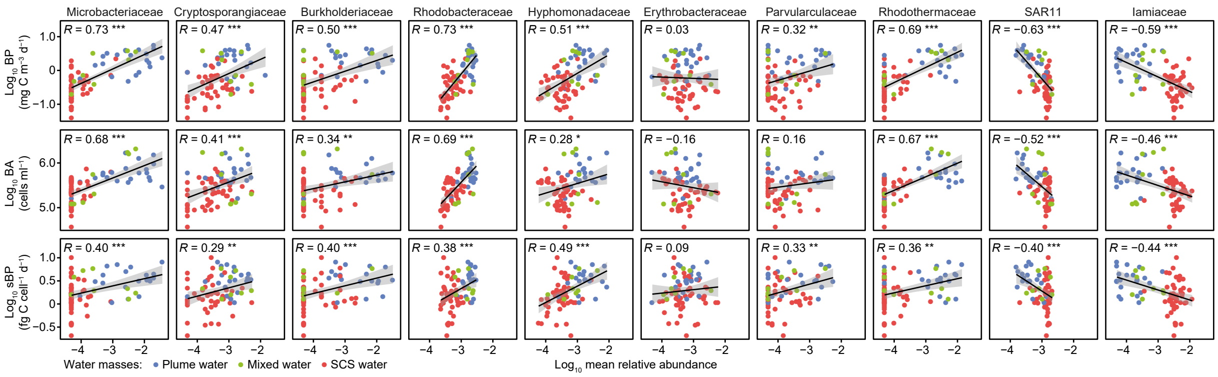

Supplementary Figure S5 Scatter plot showing the relationships between the mean relative abundance within the top 10 most abundant families and bacterial production (BP), bacterial abundance (BA), and cell-specific bacterial production (sBP). Points are colored to represent different water masses. *R* indicates the Pearson correlation coefficient. Significant levels are: **P* < 0.05, ***P* < 0.01, and ****P* < 0.001.
